# Supplementary material for: The prevalence of alcohol consumption and its related factors in adolescents: Findings from Global School-based Student Health Survey
Source: PLoS One. 2024 Apr 1;19(4):e0297225. doi: 10.1371/journal.pone.0297225 (PMC10984532; doi:10.1371/journal.pone.0297225)
Supplement: Attachment — Submitted filename: Response to Reviewers.docx [file pone.0297225.s001.docx]

***We appreciate you and the reviewers for your precious time in reviewing our paper and providing valuable comments. It was your valuable and insightful comments that led to possible improvements in the current version. The authors have carefully considered the comments and tried our best to address every one of them.***

|  | **Comment from editor** |
| --- | --- |
| The conclusion was changed in the abstract section and at the end of the text of the article. | The conclusion in the abstract is very generic, please adjust as per the conclusion in the main body of the manuscript. In addition, once you have considered the comments from one of the reviewers to reanalyze the data, please revisit the key findings of the analysis after and then try to draw specific conclusion and recommendation for the manuscript. If possible try to draw categorical recommendations for policy makers and program managers/implementers specifically unlike a general recommendation now. |

|  | **Comment from Reviewer 1** |
| --- | --- |
| The title has been changed. | The title of the MS is not clear. I would suggest revise this like "The prevalence of alcohol consumption and its related factors in adolescents: findings from Global School Health Surveys of 55 countries |
| In the whole text, students were removed and adolescents were replaced. | The authors should have used a uniform term for study participants either students or adolescents. |
| This was a descriptive cross-sectional study that was performed on … | In abstract under heading methods, it is written that "this cross-section study was performed on ..... is not correct. |
| This sentence was deleted from the text of the article. | Similarly in ethic statement, it is written that "written informed consent was obtained from each participant" which is not correct. Authors have not directly taken written consent, therefore, delete this. |
| The criteria for selecting these countries were Least Developed Countries (LDCs). | The selection criteria of 55 countries is not mentioned. |
| Given that the questions are sensitive, we felt that the response rate(RR) report would be helpful. | In routine rate response rate is given for the survey, not for a few questions and a rate above 80% is considered acceptable. Therefore, the term high and low response rate are confusing |
| Considering that no study has been done in this field before, the information in Table No. 2 can be helpful for future researchers. | The table 2 is redundant and may be deleted. |
| alcohol use indicators include (last month's use, ever drunk, two or more drinks in a day, and trouble from drinking) | In table 3, the analysis has been given as alcohol and nonalcohol. However, there is no description available in methods. |

|  | **Comment from Reviewer 2** |
| --- | --- |
| Also, During the investigations that the researchers conducted, however, this data set is old and related to 9 years ago but there is no accurate data for this country. in the investigation of research literature, it was found that the prevalence of alcohol consumption in adolescents living in countries was Least Developed Countries (LDCs) has not been widely investigated in any research, so the information of this research even if it is related to the past years but It can help researchers in the field of adolescents, therapists, and policymakers to conduct detailed investigations in this field and take basic measures in the field of prevention and treatment of alcohol in adolescents according to the important factors identified in this research. | • Authors have considered the data from 2003 to 2013 in the analysis. Authors have not provided any reasons for not including data from countries where the survey was completed after 2013. The latest dataset used in the study dates back approximately 9 years from now, and the prevalence could have substantially changed in the period. I suggest authors to include latest datasets and reanalyze it to make the findings more relevant to present context. In doing so, authors may choose to drop some of the oldest datasets from the analysis process to make sure that data are comparable across countries |
| Before comparing the countries, during the investigations, it was found that during these years, the prevalence rate within these countries did not differ striking, but there is a difference compared to other countries, for this reason, in this research, the prevalence of alcohol among the countries Less developed was investigated. | • Authors have pooled data from countries where the survey was done upto 10 years apart. So, thecomparison of prevalence of tobacco use across countries looks less logical. |
| Table 4 was changed. | Also, it would be good to clearly state the percentage of variation explained by multivariable regression model. |
