# Supplementary material for: The prevalence of alcohol consumption and its related factors in adolescents: Findings from Global School-based Student Health Survey
Source: PLoS One. 2024 Apr 1;19(4):e0297225. doi: 10.1371/journal.pone.0297225 (PMC10984532; doi:10.1371/journal.pone.0297225)
Supplement: Attachment — Submitted filename: Response to Reviewers.docx [file pone.0297225.s003.docx]

***We appreciate you and the reviewers for your precious time in reviewing our paper and providing valuable comments. Your valuable and insightful comments led to possible improvements in the current version. The authors have carefully considered the comments and tried our best to address every one of them.***

|  | **Comment from editor** |
| --- | --- |
| All items have been changed. | Revised title: the correct name of GSHS is Global School-based Student Health Survey. Kindly use the right one. Abbreviated form in title may not be required.  Abstract: The data source being GSHS is not explicitly mentioned. Mention that in the abstract. Introduction: the line mentioning objective at the end of the introduction is replaced with the new title of the objective and this does not look appropriate. There was no need to change it this way. The previously mentioned objective was fine.  Methods: GSHS is not provided with full form in its first use in the main body of the manuscript. Please review the manuscript thoroughly one more time, for any other such errors.  LDCs was mentioned as the country selection criteria, however, the countries listed in the study do not match the list as per the UN classification of LDCs (https://www.un.org/ohrlls/content/profiles-ldcs). Include citation for LDCs in the manuscript. A better option to select countries would be to follow the world bank classification of countries by income level. But it’s up to authors to decide.  In addition, the authors have provided clarification for non-inclusion of dataset beyond 2013 as non-availability of the data. But the datasets for some LDCs (some were verified by the editor) are available beyond 2013 as well (https://extranet.who.int/ncdsmicrodata/index.php/catalog/GSHS). The link provided by the authors is not the right one for dataset, that is for the questionnaires. Not sure from where you accessed the dataset. Include citation for the source of dataset in the manuscript. With this, authors are advised to add the newer datasets to the analysis which makes the manuscript more relevant to the present context. We believe you definitely have the codes of data cleaning and analysis stored; hence it should not take long time to reanalyze. Once you submit the revised version, your manuscript will be given top priority for review on time, considering the time consumed in rework of this important manuscript. Hence, authors are requested to re-consider the comment by reviewer 2 about the datasets by time. |

|  | **Comment from Reviewer 1** |
| --- | --- |
| The title has been changed. | The title of the MS is not clear. I would suggest revise this like "The prevalence of alcohol consumption and its related factors in adolescents: findings from Global School Health Surveys of 55 countries |
| In the whole text, students were removed and adolescents were replaced. | The authors should have used a uniform term for study participants either students or adolescents. |
| This was a descriptive cross-sectional study that was performed on … | In abstract under heading methods, it is written that "this cross-section study was performed on ..... is not correct. |
| This sentence was deleted from the text of the article. | Similarly in ethic statement, it is written that "written informed consent was obtained from each participant" which is not correct. Authors have not directly taken written consent, therefore, delete this. |
| This study was conducted on data collected from low- and middle-income countries between 2003 and 2018, by World Health Organization guidelines. | The selection criteria of 55 countries is not mentioned. |
| Given that the questions are sensitive, we felt that the response rate(RR) report would be helpful. | In routine rate response rate is given for the survey, not for a few questions and a rate above 80% is considered acceptable. Therefore, the term high and low response rate are confusing |
| Considering that no study has been done in this field before, the information in Table No. 2 can be helpful for future researchers. | The table 2 is redundant and may be deleted. |
| alcohol use indicators include (last month's use, ever drunk, two or more drinks in a day, and trouble from drinking) | In table 3, the analysis has been given as alcohol and nonalcohol. However, there is no description available in methods. |

|  | **Comment from Reviewer 2** |
| --- | --- |
| Data from recent years that were available were added. | • Authors have considered the data from 2003 to 2013 in the analysis. Authors have not provided any reasons for not including data from countries where the survey was completed after 2013. The latest dataset used in the study dates back approximately 9 years from now, and the prevalence could have substantially changed in the period. I suggest authors to include latest datasets and reanalyze it to make the findings more relevant to present context. In doing so, authors may choose to drop some of the oldest datasets from the analysis process to make sure that data are comparable across countries |
| Before comparing the countries, during the investigations, it was found that during these years, the prevalence rate within these countries did not differ striking, but there is a difference compared to other countries, for this reason, in this research, the prevalence of alcohol among the countries Less developed was investigated. | • Authors have pooled data from countries where the survey was done upto 10 years apart. So, thecomparison of prevalence of tobacco use across countries looks less logical. |
| Table 4 was changed. | Also, it would be good to clearly state the percentage of variation explained by multivariable regression model. |
